# Supplementary material for: Diverse Members of the Phylum Armatimonadota Promote the Growth of Aquatic Plants, Duckweeds
Source: Int J Mol Sci. 2025 Oct 9;26(19):9824. doi: 10.3390/ijms26199824 (PMC12524902; doi:10.3390/ijms26199824)
Supplement: Supplementary file 1 [file ijms-26-09824-s001.zip › ijms-3908202-supplementary.pdf]

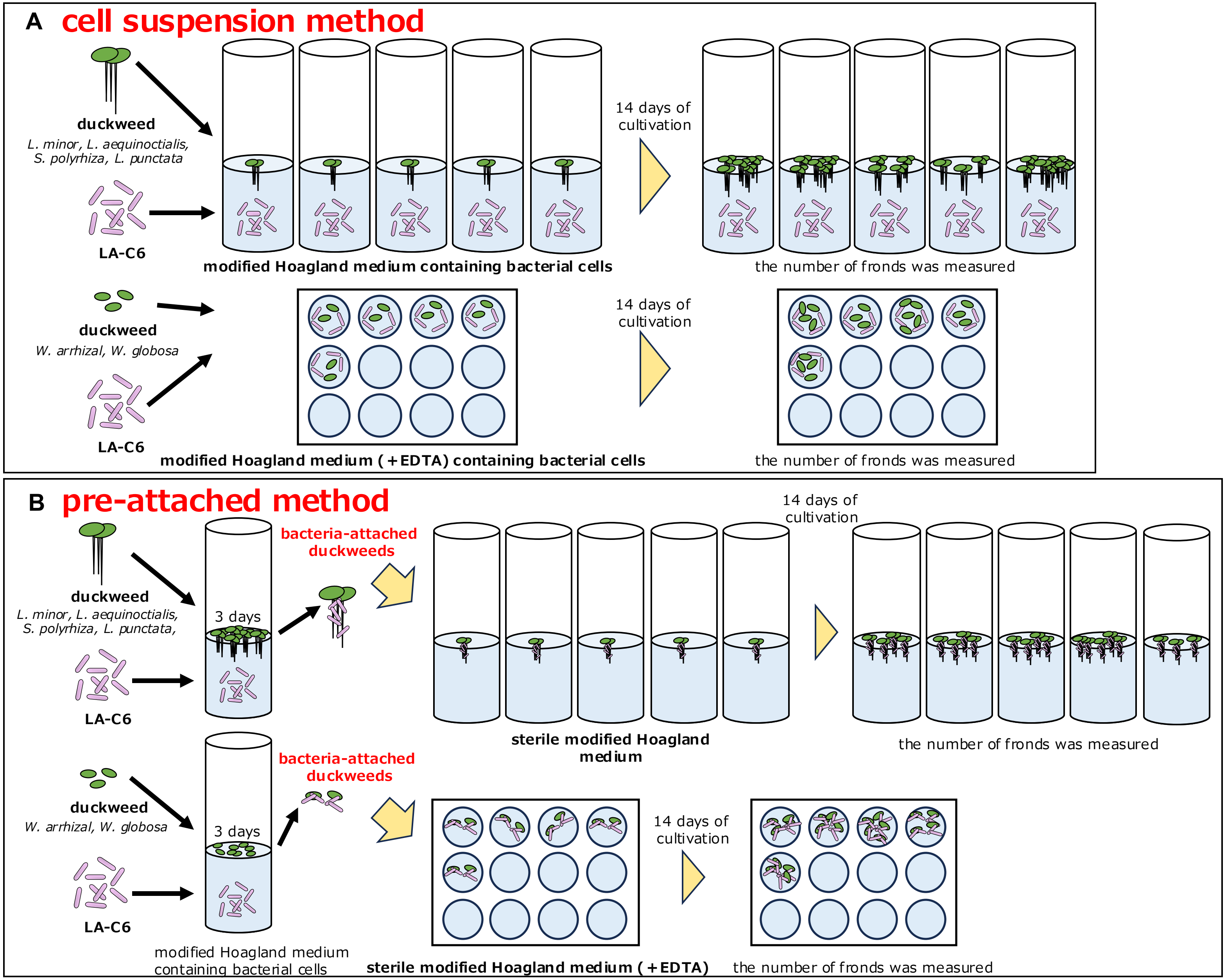

Figure S1. Schematic diagram of experiments evaluating the plant growth-promoting (PGP) effect of strain LA-C6. (A) PGP effect on aseptic duckweeds soaked in modified Hoagland medium containing bacterial cells (cell suspension method). (B) PGP effect on duckweeds pre-attached with bacteria and cultivated in sterile modified Hoagland medium (pre-attached method).

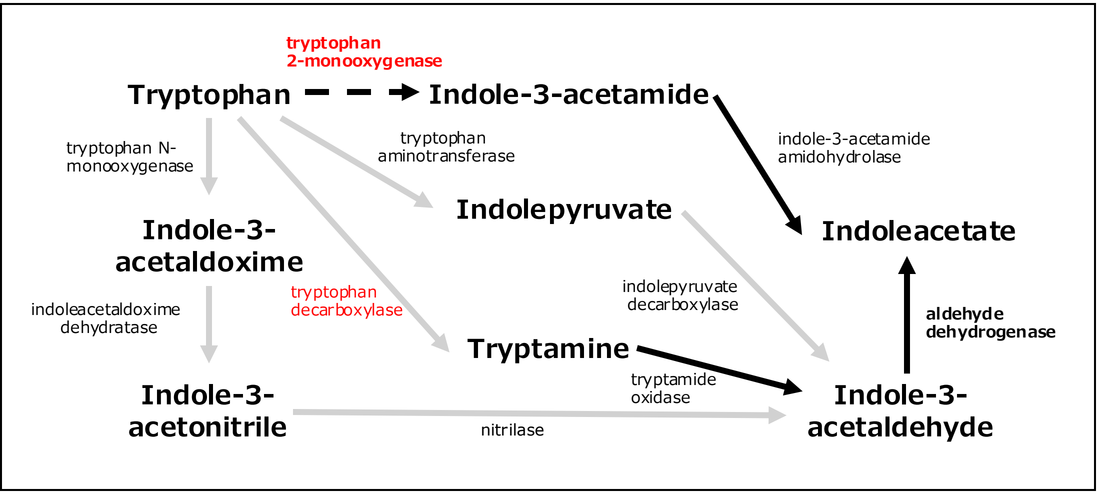

Prediction of tryptophan 2-monooxygenase [EC1.13.12.3]

The genomic sequence of strain LA-C6 (4091 seqs) was searched by blastp using this enzyme-related gene as a query.

No enzyme with a matching name was detected. An ORF (LA-C6_00806) was the most closely related sequence was L-amino acid dehydrogenase (E-value=2×10^{-7}, 24% identity).

An ORF detected as the most closely related sequence was searched for protein domains by using Pfam.

This sequence contained domains that was classified into a group including tryptophan 2-monooxygenase.

Prediction of tryptophan decarboxylase [EC4.1.1.28 or EC4.1.1.105]

No closely related genes were detected in strain LA-C6 genome by using blastp and Pfam analyses.

Figure S2. Prediction of IAA synthesis pathway of strain LA-C6. The map was generated by KEGG mapper using a KO file as a query. Detailed gene prediction was performed using Pfam. Black and gray line arrows indicate predicted and non-predicted reactions for the strain, respectively. A dotted line arrow indicates manually predicted genes.

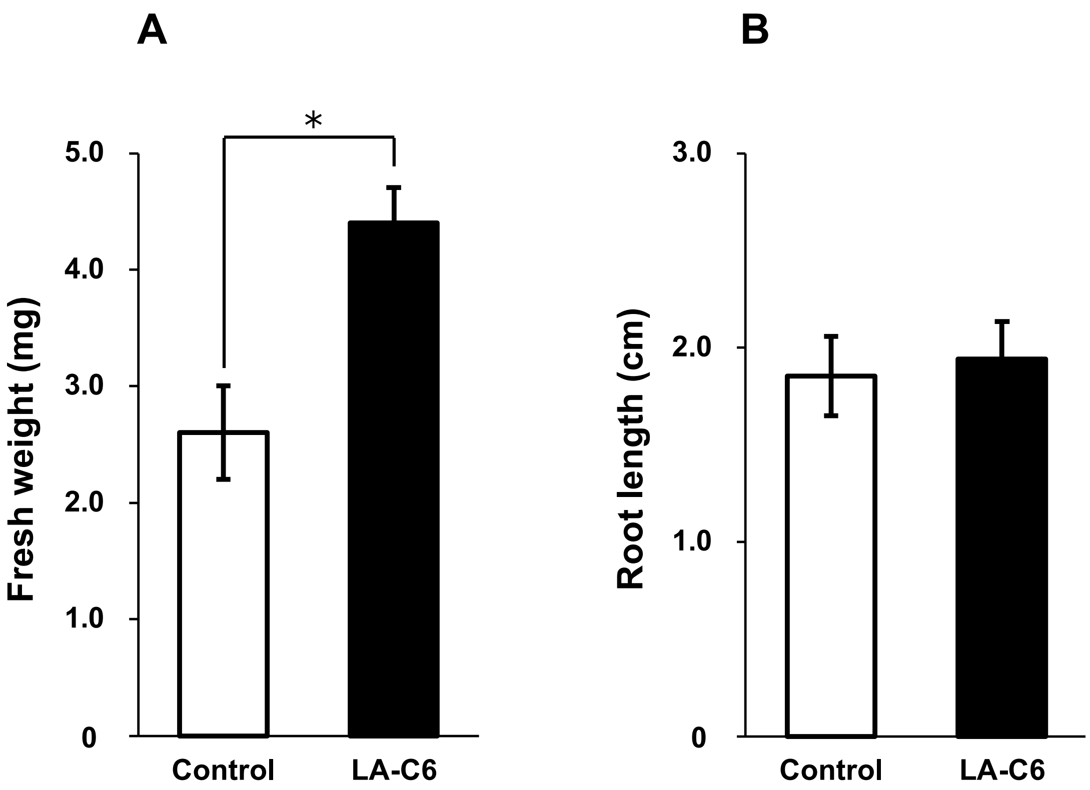

Figure S3. Plant growth-promoting effects of strain LA-C6 on *Arabidopsis thaliana* seedlings. (**A**) The fresh weight and (**B**) the root length of *Arabidopsis thaliana* after 22 days of cultivation. $n = 3$; *, $p < 0.05$, error bars represent SE.
